# Aberrant S293 Phosphorylation Drives Oligomerization of Tau Repeat R2: Insights from Molecular Dynamics Simulations

**DOI:** 10.1021/acschemneuro.5c00734

**Published:** 2025-10-23

**Authors:** Viet Hoang Man, Xibing He, Phuong H. Nguyen, Jie Gao, Junmei Wang

**Affiliations:** 1 Department of Pharmaceutical Sciences and Computational Chemical Genomics Screening Center, School of Pharmacy, 6614 University of Pittsburgh, Pittsburgh, Pennsylvania 15261, United States; 2 Laboratoire de Biochimie Theorique UPR 9080, CNRS, Université Denis Diderot, Sorbonne Paris Cité IBPC, 13 Rue Pierre et Marie Curie, Paris 75005, France; 3 Department of Neuroscience, 555039 The Ohio State University Wexner Medical Center, Columbus, Ohio 43210, United States

**Keywords:** Tau repeats, oligomerization, phosphorylation, S293, REMD, simulation

## Abstract

Aberrant phosphorylation, which is absent in healthy brains but present exclusively in the brains of individuals with Alzheimer’s disease (AD), plays a critical role in AD development. It causes the dissociation of tau protein from microtubules, followed by the aggregation of tau protein into brain-toxic oligomers and fibrils. In our previous study, we investigated the impact of abnormal phosphorylation at S289 (pS289) on the oligomerization of tau repeat R2 peptides. In this work, we continue to investigate the effect of aberrant phosphorylation at residue S293 (pS293) on the R2 peptides. Our result indicated that pS293 also promotes oligomerization, which is similar to pS289. Both the phosphorylation-enhanced intramolecular and intermolecular interactions and β-sheet formation of phosphorylated R2 compared to that of the wild type. We observed that Na^+^ can bridge two pS293 residues to form pS293--Na^+^-pS293 triad in the R2 dimer, a phenomenon also observed for the pS289 R2 dimer. However, the impact of pS293 was different from that of pS289 in terms of the secondary structural profile of both monomeric and dimeric R2 peptides. Our findings suggest that phosphorylation at S293 should be taken into consideration in the inhibitor screening of tau oligomerization.

## Graphical abstract

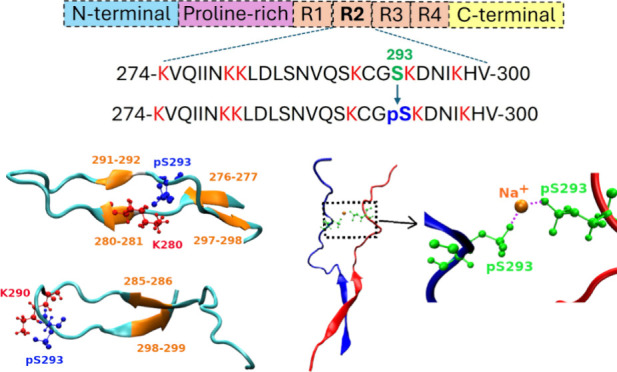

## Introduction

Alzheimer’s disease (AD) is the most common cause of dementia. AD patients have experiences with memory loss, disorientation, loss of motivation, language decay, difficulty in managing self-care, and behavioral issues.^1^ Therefore, it consumes a high cost to care for AD patients. The number of people with dementia in the world was 57.4 million in 2019, and it is estimated to reach 152.8 million in 2050.^2^ Currently, no cure or effective treatment for AD is available yet. AD is also known as one of the most common tauopathies that are associated with the loss function of tau protein. In a healthy brain, tau protein plays some important physiological roles, such as stabilizing and bundling axonal microtubules (MTs). It regulates MTs through a phosphorylated–dephosphorylated reversible process. However, phosphorylation at specific residues, naming abnormal phosphorylation, can make tau lose affinity to MTs and form aggregates. Tau aggregation produces toxic brain agents, including soluble oligomers and insoluble fibrils. The fibrils, paired helical filaments (PHFs), are the main components of insoluble neurofibrillary tangles (NFTs), which are a hallmark of neurodegenerative tauopathies, disrupt normal cellular functioning, and result in the death of the nerve cells.^3^ Tau oligomers (TOs), formed in the early stage of tau aggregation, cause neuronal damage, leading to neurodegeneration and traumatic brain injury.^4–6^ Recent evidence shows soluble TOs are more toxic than insoluble NFTs.^7–9^ Therefore, investigating the effects of abnormal phosphorylation on tau oligomerization is highly intriguing.

Tau exhibits six isoforms, ranging in length from 352 to 441 residues. A full-length adult tau (441 residues) comprises an N-terminal projection, a proline-rich domain, a microtubule (MT) binding region, and a C-terminal domain. The N-terminal and C-terminal domains display significant disorder, while the MT-binding region is more structured.^10^ The MT-binding region contains three or four imperfect sequence repeats, R1, R2, R3, and R4. These repeats play crucial roles not only in MT-binding but also in the pathological aggregation of Tau proteins.^11–13^ The full-length tau contains 85 potential phosphorylation sites, encompassing 45 serine, 35 threonine, and 5 tyrosine residues. Among these, approximately 20 residues undergo phosphorylation in tau from both healthy and AD brains, while about 40 ones occur exclusively in the tau of AD brains.^14^ The diverse phosphorylation sites and their combinations yield varied effects on tau aggregation. For instance, phosphorylation at T175 ^15^ or triple phosphorylation at S202/T205/S208 ^16^ can accelerate tau aggregation, while some phosphorylation sites such as S214, S262, and S305 inhibit tau aggregation.^17,18^ Pseudophosphorylation at T17, T212,^19^ and S202/T205 ^20^ facilitates tau filament formation, while S422 ^21^ pseudophosphorylation enhances tau oligomerization. Although many studies have performed to investigate the impact of aberrant phosphorylation on tau aggregation,^15–21^ there are still many abnormal phosphorylation sites having not been characterized yet.

Molecular dynamics (MD) simulations play a crucial role in studying amyloid aggregation.^22^ They are widely employed to investigate the oligomerization of amyloid and to characterize the structures of soluble amyloid oligomers at the atomic level—a challenge that current experimental technologies struggle to address.^23–29^ Some enhanced sampling methods such as replica exchange molecular dynamics (REMD) have been developed to accelerate the exploration of free-energy landscapes. However, conducting an MD simulation study for the oligomerization of full-length tau proteins is currently computationally intensive due to the large size and disordered nature of the proteins. Thus, tau fragments such as tau repeats and PHF6 (^306^VQIVYK^311^) and PHF6* (^275^VQIINK^280^) peptides are usually utilized since they are essential for tau fibrillization and are considered as surrogates of the full-length tau.^10,29–37^ In our recent work, we performed intensive REMD simulations to investigate the impact of phosphorylated S289 on the oligomerization of tau R2 peptides (274–300 residues).^29^ Our result indicated that abnormal phosphorylation at the S289 residue (pS289) can enhance the oligomerization of tau R2 peptides by promoting intermolecular interaction. In this work, we applied similar computational approaches to examine the effect of another abnormal phosphorylation at the S293 residue (pS293) on the oligomerization of tau R2 peptides. We found that the pS293 not only increases intermolecular interaction but also promotes β-sheet formation of R2 peptides. Therefore, it accelerates the oligomerization of the R2 peptides. Note that pS293 is one of the abnormal 40 phosphorylation sites,^14^ and it has been determined in insoluble PHF from AD brain.^38^ Our finding may reveal the critical role of pS293 in AD.

## Results

In our previous work studying the impact of phosphorylation at SER289 (pS289) on the oligomerization of R2 peptides, we have conducted REMD simulations from diverse initial structures.^29^ This approach not only provided the convergence of conformational sampling but also covered a larger amount of structural space. In this work, we applied the same protocol to investigate the effect of abnormal phosphorylation at S293 (pS293) on the oligomerization of the R2 peptides. To evaluate the convergence of simulations, we first analyzed the time dependence of various peptide properties including root-mean-square deviation (RMSD), gyration of radius (*R*_g_), solvent-accessible area surface (SASA), and β-content at 311 K for the monomeric systems and 309.4 K for the dimeric systems (Figures S1–S3 in Supporting Information). The results showed that those structural parameters fluctuated around equilibrium values during simulation time, indicating that wide conformational spaces of R2 monomers and dimers were sampled. This also confirmed that the intrinsically disordered nature of the tau fragments was well captured. Next, we examined two ensemble statistics of structural parameters, including *R*_g_, SASA, and number of intermolecular/intramolecular interactions. As indicated by Figure S4 in Supporting Information, excellent convergence was achieved for both the monomeric and dimeric systems as the distributions from the two statistics were essentially similar. The secondary structures, *R*_g_, and SASA shown on Figure S5 (for monomeric systems) and Figure S6 (for dimeric systems) in SI demonstrated a similar trend of the R2 monomeric or dimeric systems across a wide range of replica temperatures. Thus, for the rest of the text, unless mentioned explicitly, all the results are ensemble statistics at 311 K and within the simulation time of 100–300 ns for the monomeric systems and 309.4 K and within the simulation time of 100–500 ns for the dimeric systems.

### Phosphorylation at S293 Residue Promotes Intramolecular Interaction and β-Sheet Formation of the R2 Peptide

To examine the impact of pS293 on the structure of monomeric R2 peptides, we first considered the overall structural parameters, including SASA and *R*_g_. The distributions of those parameters are shown in Figure 1, and their average values are listed in Table 1. The data indicated that the phosphorylated peptides were more compact than those of the wild type. For both *R*_g_ and SASA, the distribution peaks were located at smaller values in the pS293R2 system than the corresponding ones in wild-type R2 (wtR2) system. The average values of pS293R2 peptides, 1.2 nm *R*_g_ and 31.6 nm^2^ for SASA, were significantly smaller than the corresponding values of wtR2 peptides, which were 1.36 and 32.6 nm^2^. The compactness results implied pS293R2 peptides have stronger intramolecular interactions than wtR2 peptides.

**Table 1:** Average of Overall Structural Parameters of Monomer wtR2 and pS293R2 Peptides^a^

system	β	helix	turn	coil	SASA	*R*_g_	*N*_intra‑SC_	*N*_intra‑HB_
**wtR2**	7.9	2.8	30.2	59.0	32.6	1.36	16.75	2.30
**pS293R2**	11.5	1.8	30.7	56.0	31.6	1.26	20.02	2.62

a The parameters include secondary structures in % (β, helix, turn, and coil contents), solvent-accessible surface area in nm^2^ (SASA), gyration radius in nm (*R*_g_), number of intramolecular side chain–side chain interaction (*N*_intra‑SC_), and number of intramolecular hydrogen bond interaction (*N*_intra‑HB_).

**Figure 1:**
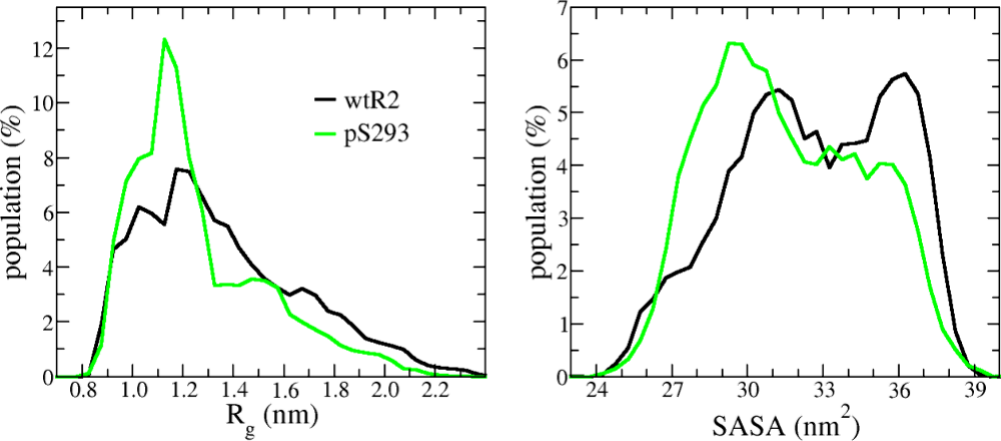
The distributions of gyration radius (*R*_g_) and solvent-accessible surface area (SASA) for wtR2 (black) and pS293R2 peptides.

To directly compare intramolecular interactions between wtR2 and pS293R2, we further performed an intramolecular residue–residue interaction analysis. As shown in Figure 2, phosphorylation at Ser293 promoted the numbers and frequencies of both intramolecular side chain–side chain and hydrogen bond interactions of R2 peptides. Compared to those of the wild type, the average numbers of the side chain–side chain and hydrogen bond interactions of the phosphorylated peptide increased 20 and 14%, respectively (Table 1). The interaction maps showed that the phosphorylation enhanced the interactions between N-terminal residues (274–284) and C-terminal residues (290–300) as well as between C-terminal residues (Figure 2). Interestingly, the side chain–side chain interactions of the pS293 residue with K280–K281 and K298 residues in the pS293R2 peptide are much stronger than those of the unphosphorylated S293 residue with K280–K281, K290, and K298 residues in the wtR2 peptide (Figure 2a,c). This result can be explained by that the phosphorylated-S293 carries negative charges with positive-charged residues including K280, K281, K290, and K298. The strong side chain–side chain interaction between pS293 residue and the lysine residues also result in more hydrogen bonds formed with their neighbor residues as well as hairpin formation. In particular, enhanced hydrogen bonding was observed between the following residue pairs: 280–291, 281–291, 282–292, 290–298, 292–296, and 293–296 (Figures 2b,d). The interaction between K280–K281 and pS293 also contributes to the increased level of hydrogen bonding between N-terminal and C-terminal residues (Figure 2b,d).

**Figure 2:**
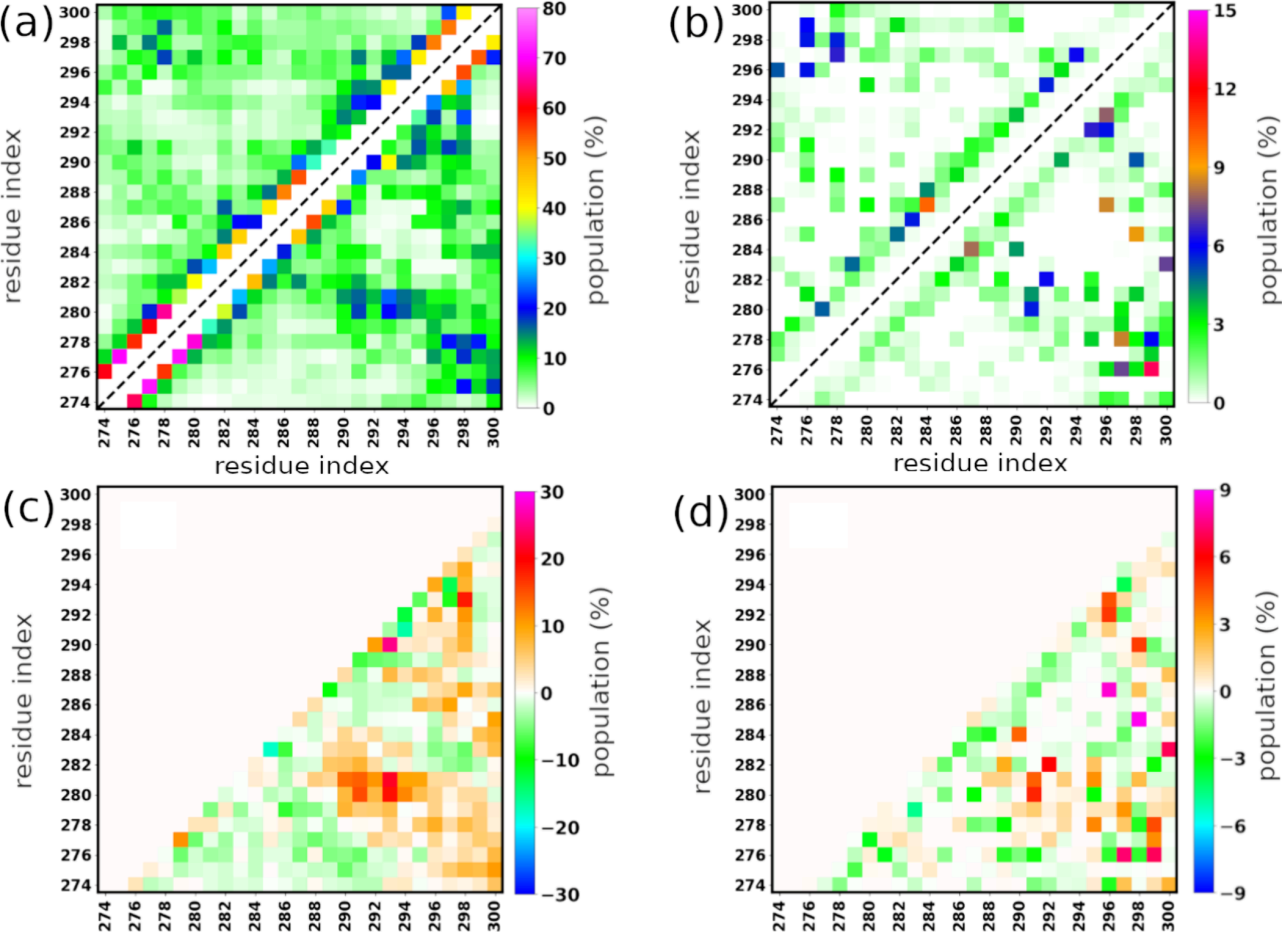
Intramolecular residue–residue interactions. The interactions were characterized using multiple metrics: side chain–side chain contact maps (a), hydrogen bond maps (b), and the differences in side chain–side chain interactions (c) and hydrogen bonds (d) between pS293R2 and wtR2 peptides. In panels (a) and (b), the upper-left and lower-right triangles represent the wtR2 and pS293R2 peptides, respectively.

Next, we investigated the impact of phosphorylation on the secondary structure of the monomeric R2 peptide. The average secondary structural contents of wtR2 and pS293R2 peptides are listed in Table 1. It shows that the β content of pS293R2 is significantly greater than that of wtR2, while the helix and coil contents of pS293R2 are slightly smaller than those of wtR2. The turn contents of pS293R2 and wtR2 are similar. To study each residue’s ability to form certain types of secondary structure, we also analyzed the secondary structural profile along the sequence. As shown in Figure 3, the turn profiles of pS293R2 and wtR2 were almost identical, while the coil and β profiles of the two peptides were significantly different at C-terminal residues, and the helix profiles of the two peptides were different for most residues. The helix propensity of residues 274–291 in the wtR2 peptide was slightly higher than in the pS293R2 peptide, whereas the helix propensity of residues 293–297 was lower in the wtR2 peptide compared to that in the pS293R2 peptide. The phosphorylation enhanced the β-propensity of residues 280–281, 284–286, and the C-terminal residues 296–299. To further examine how phosphorylation influences β-structure, we analyzed residue–residue β-sheet formation (Figure 4). The results showed that phosphorylation markedly enhanced β-sheet formation between the following residue pairs: 276–297, 276–299, 277–299, 280–291, 280–297, 285–298, 290–298, and 292–296. These findings were consistent with the observed increases in side chain–side chain interactions and residue–residue hydrogen bonding. Moreover, they explained the increased β-propensity of residues 280–281, 284–286, and 296–299 and indicated that the structural effect of phosphorylation propagates across a broader segment of the C-terminal region (Figure 4c,d,e).

**Figure 3:**
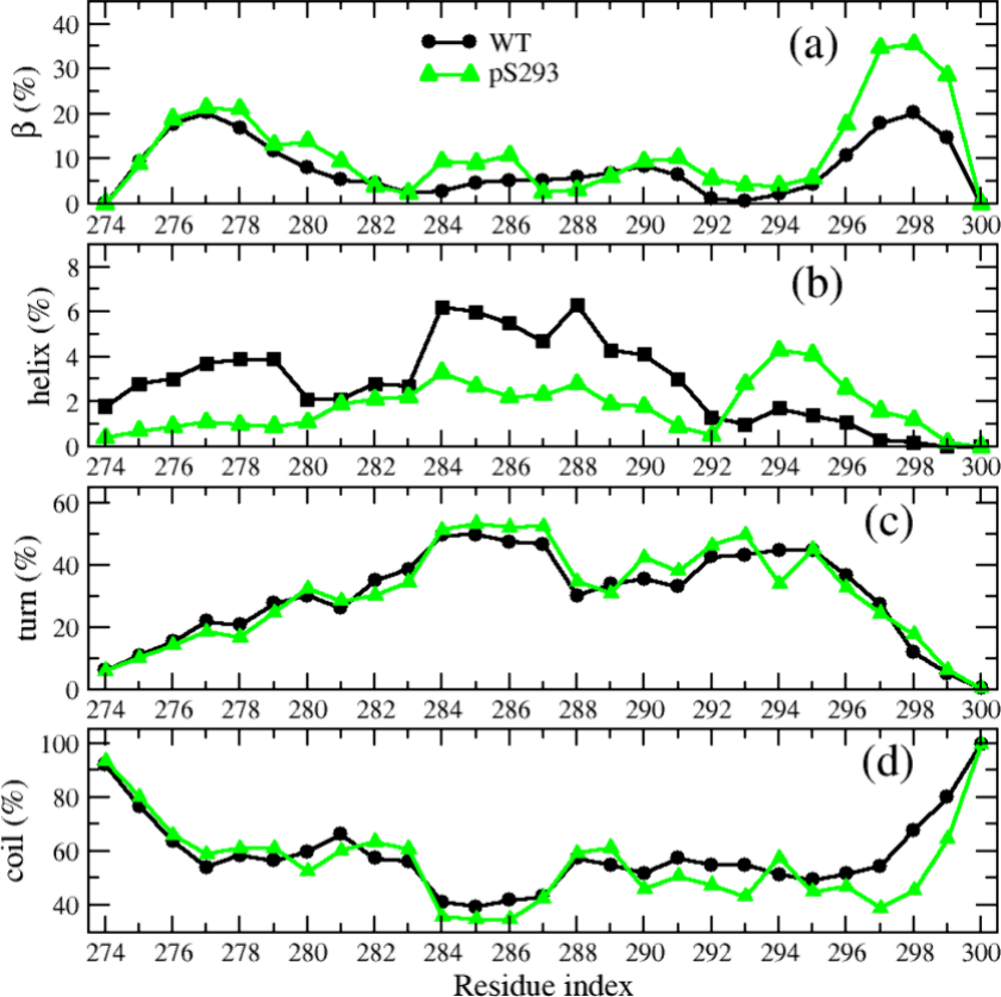
Secondary structural propensities along amino acid sequence of wtR2 (black) and pS293R2 (green) peptides.

**Figure 4:**
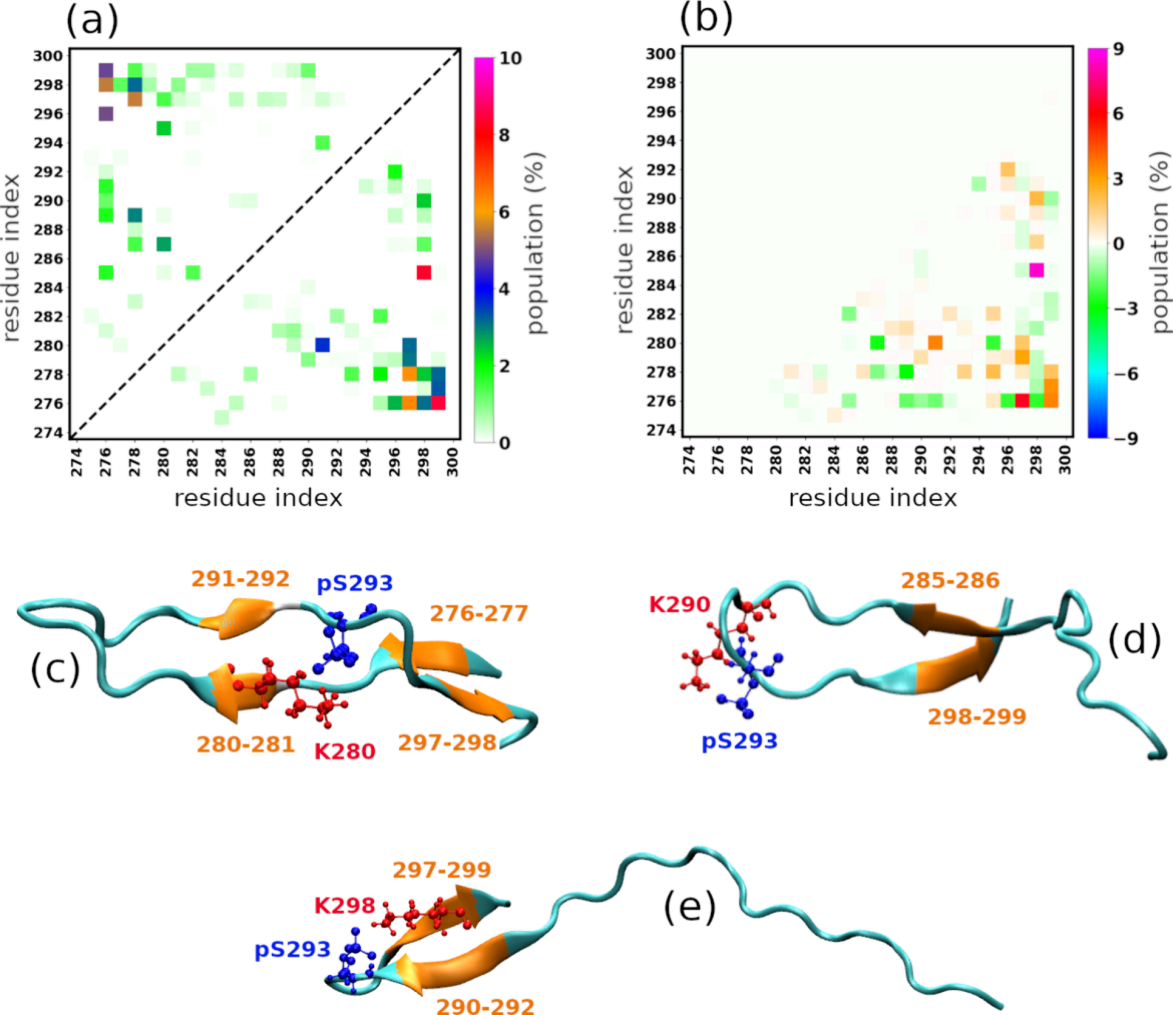
Intramolecular residue–residue β-sheet formation. In panel (a), the upper-left and lower-right triangles represent the wtR2 and pS293R2 peptides, respectively. Panel (b) is the difference of the residue–residue β-sheet formation between pS293R2 and wtR2 peptides. Panels (c), (d), and (e) are representative pS293R2 structures, which show strong interactions between pS293 residue and K residues. The pS293-K interactions result in β-sheet formations. In panels (c), (d), and (e), the pS293 residue is represented in blue, K residue in red, and residues of β-sheets in orange.

### Phosphorylation at the S293 Residue Enhances Intermolecular Interaction of the R2 Peptide

In this part, we examine the impact of phosphorylation on the intermolecular interaction of R2 peptides, which plays an important role in oligomerization. Figure 5 shows the intermolecular side chain–side chain (sci) and hydrogen bond (hbi) interaction maps as well as their interaction frequency of individual residues in three dimeric systems, wtR2+wtR2, wtR2+pS293R2, and pS293R2+pS293R2. The total numbers of side chain–side chain and hydrogen bond interactions ascend from wtR2+wtR2 to wtR2+pS293R2 to pS293R2+pS293R2, while SASA and *R*_*g*_ decrease sequentially with the same order of sequence. This result indicates that the intermolecular interaction is proportional to the compactness of the dimers (Table 2). Our data showed that phosphorylation at S293 not only enhances the intermolecular interaction but also changes the pattern of the interaction maps. In the wtR2+wtR2 system, interactions commonly took place between the middle residues of the first peptide and those of the second peptide, the N-terminal residues of one peptide and the C-terminal residues of the other, and the N-terminal residues of one peptide and the middle residues of the other. Strong interaction occurred at residue pairs 276–286, 277–282, 277–284, 278–292, 282–284, 284–284, and 284–284. In the wtR2+pS293R2 system, interaction frequently occurred between N-terminal residues of both peptides, N-terminal residues of the wild-type peptide, and middle residues of the phosphorylated peptide. Different from the wtR2+wtR2 case, the pS293 residue of the phosphorated peptide strongly interacted with K280, K281, K290, and K298 residues of the wild-type peptide. These strong interactions stem from the opposing net charges of lysine and phosphorylated serine. In the pS293R2+pS293R2 system, strong interactions occurred between the PHF6* residues of two peptides, 289–294 residues of two peptides, and 280–284 residues of one peptide and 289–295 residues of the other peptide. Intriguingly, strong interactions were also found between two phosphorylated residue regions (pS293-K294) of the phosphorylated peptides.

**Table 2:** Average of Overall Structural Parameters of Dimeric wtR2+wtR2, wtR2+pS293R2, and pS293R2+pS293R2 Peptides^a^

System	β	helix	turn	coil	SASA	*R*_g_	*N*_inter‑SC_	*N*_inter‑HB_
**wtR2+wtR2**	14.2	1.9	26.2	57.8	59.94	1.79	24.53	3.12
**wtR2+pS293R2**	16.0	2.1	25.9	56.0	58.20	1.67	27.05	3.40
**pS293R2+pS293R2**	15.2	2.2	29.2	53.4	56.61	1.55	32.85	3.75
**KpS293R2+pS293R2**	17.7	1.6	27.8	53.1	56.12	1.58	32.97	4.07

a The parameters include secondary structures in % (β, helix, turn, and coil contents), solvent-accessible surface area in nm^2^ (SASA), gyration radius in nm (*R*_g_), number of intermolecular side chain–side chain interaction (*N*_inter‑SC_), and number of intermolecular hydrogen bond interaction (*N*_inter‑HB_).

**Figure 5:**
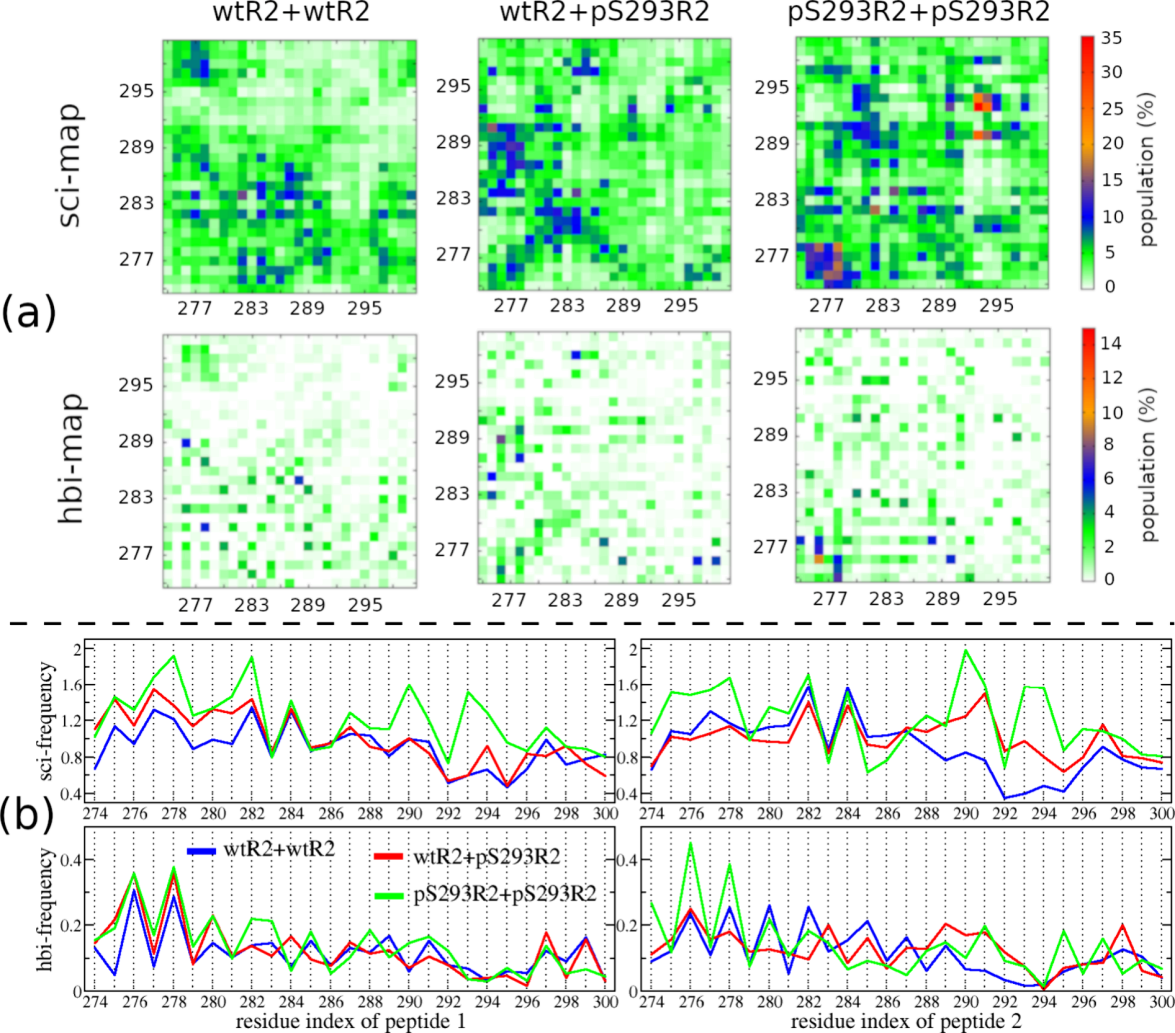
Intermolecular residue–residue interactions. (a) The upper panels show side chain–side chain interaction maps (sci-map), and the lower panels show hydrogen bond interaction maps (hbi-map). Color bars on the right indicate the interaction intensity corresponding to the maps aligned with them. (b) The upper panels display side chain–side chain interaction frequencies (sci-frequency), and the lower panels display hydrogen bond interaction frequencies (hbi-frequency), plotted along the residues of the first peptide (left panels) and the second peptide (right panels). In panel (b), blue lines are for the data from wtR2+wtR2 system, red lines for the data from the wtR2+pS293R2 system, and green lines for the data from pS293R2+pS293R2 system.

### Impact of Phosphorylation at the S293 Residue on Secondary Structures of R2 Dimers

The ensemble statistics of the secondary structures of the R2 dimers are summarized in Table 2. Our data indicates that phosphorylation at residue S293 slightly alters the overall secondary structure of R2 dimers. The β-sheet contents, 16% for the wtR2+pS293R2 dimer and 15.2% for the pS293R2+pS293R2 dimer, are both higher than that of the wtR2+wtR2 dimer. Although the average helical content of all the three dimers remains low (≈2%) globally, the residue-specific analysis (Figure 6) reveals a distinct local effect: residues near pS293 in the pS293R2+pS293R2 dimer exhibit a significant (>5%) increase in helical propensity compared with the corresponding residues in wtR2+wtR2 and wtR2+pS293R2 dimers. Specifically, in the mixed wtR2+pS293R2 system, the phosphorylated peptide chain does not display this enhanced helical propensity effect, having similar values of wtR2. Notably, the helical content increase near pS293 is also evident in the monomeric pS293R2 system, suggesting that this effect is intrinsic to the phosphorylated peptide but being amplified only when both peptide chains are phosphorylated. The effect of pS293 on the helix propensity of its neighboring residues differs from that of phosphorylation at S289 reported in our previous study, where phosphorylation decreased the helical propensity of adjacent residues.^29^ In addition, the turn content of wtR2+wtR2 and wtR2+pS293R2 dimers is ∼26%, notably lower than the 29.2% observed in the pS293R2+pS293R2 dimers. The coil content progressively decreases from wtR2+wtR2 to wtR2+pS293R2 to pS293R2+pS293R2. Together, these results suggest that phosphorylation at S293 supports a disordered-to-ordered transition of tau R2 peptides with both enhanced β-sheet formation and localized helical stabilization contributing to altered structural preferences. Importantly, the absence of a helix increase in the mixed dimer highlights that the effect is cooperative and depends on the phosphorylation state of both partners. Such cooperative stabilization may represent a molecular mechanism by which multisite phosphorylation promotes conformational transitions that facilitate pathological tau aggregation.

**Figure 6:**
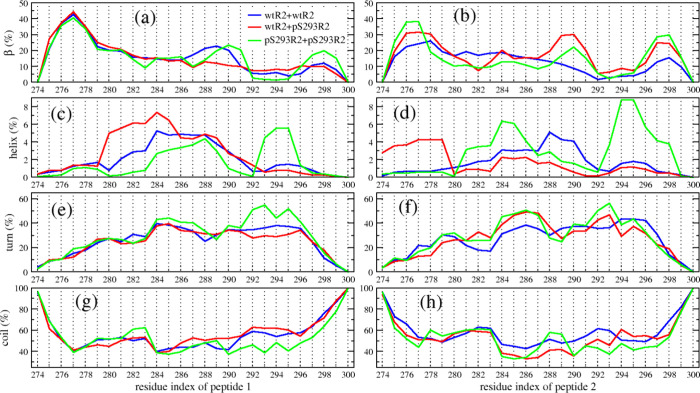
Secondary structural propensities along residues of the tau R2 peptides in wtR2+wtR2 (blue), wtR2+pS293R2 (red), and pS293R2+pS293R2 (green) systems. The propensities include β (a,b), helix (c,d), turn (e,f), and coil (g,h). The left panels are for the first peptide, and the right panels are for the second peptide in the dimeric systems.

### Role of Cations on the Oligomerization of Phosphorylated-S293 R2 Peptides

Ions play an important role in amyloid aggregation.^39^ In our simulation, a salt concentration of 0.15 M is applied to all systems, resulting in a certain amount of sodium and chloride ions present in the systems. In this part, we consider the interaction between the ions and tau R2 peptides. A cutoff of 0.3 nm is used to determine whether an interaction is formed between an ion and a residue. Figure 7 shows the interaction frequency of ions and residues of R2 peptides in the dimeric systems. As observed, sodium had strong interactions with phosphorylated-S293 residues and chloride frequently interacted with Lys residues. This result is expected since pS293 has a −2 net charge, and Lys has a +1 net charge. The interaction frequencies of sodium and the pS293 residue are around 30% in wtR2+pS293R2 and 50% in pS293R2+pS293R2, while fewer sodium–S293 interactions are found in wtR2+wtR2. The interaction frequency of chloride ion and Lys residues in wtR2+wtR2 is around 8–9%, higher than the corresponding values in wtR2+pS293R2 (5–6%) and pS293R2+pS293R2 (3–6%). Notably, the interaction frequency of sodium-pS293 is much higher than that of chloride-Lys. We further analyze the population of minimum ion-S293/pS293 distance in the dimeric systems (Figure S7). As seen, the Na^+^-S293 and Na^+^-pS293 distances are vastly different, while the populations of Cl-S293 and Cl-pS293 distances are similar. The highest peaks of Na^+^-pS293 and Na^+^-S293 distance populations are located around 2–3 and 9–18 Å, respectively. Interestingly, Na^+^-pS293 interaction in pS293R2+pS293R2 is more frequent than that one in wtR2+pS293R2, resulting in the formation of pS293-Na^+^-pS293 bridge (Figure 8). The frequency of pS293-Na^+^-pS293 bridge formation is 23.4% of the population sampled in MD simulations for the pS293R2+pS293R2 dimeric system.

**Figure 7:**
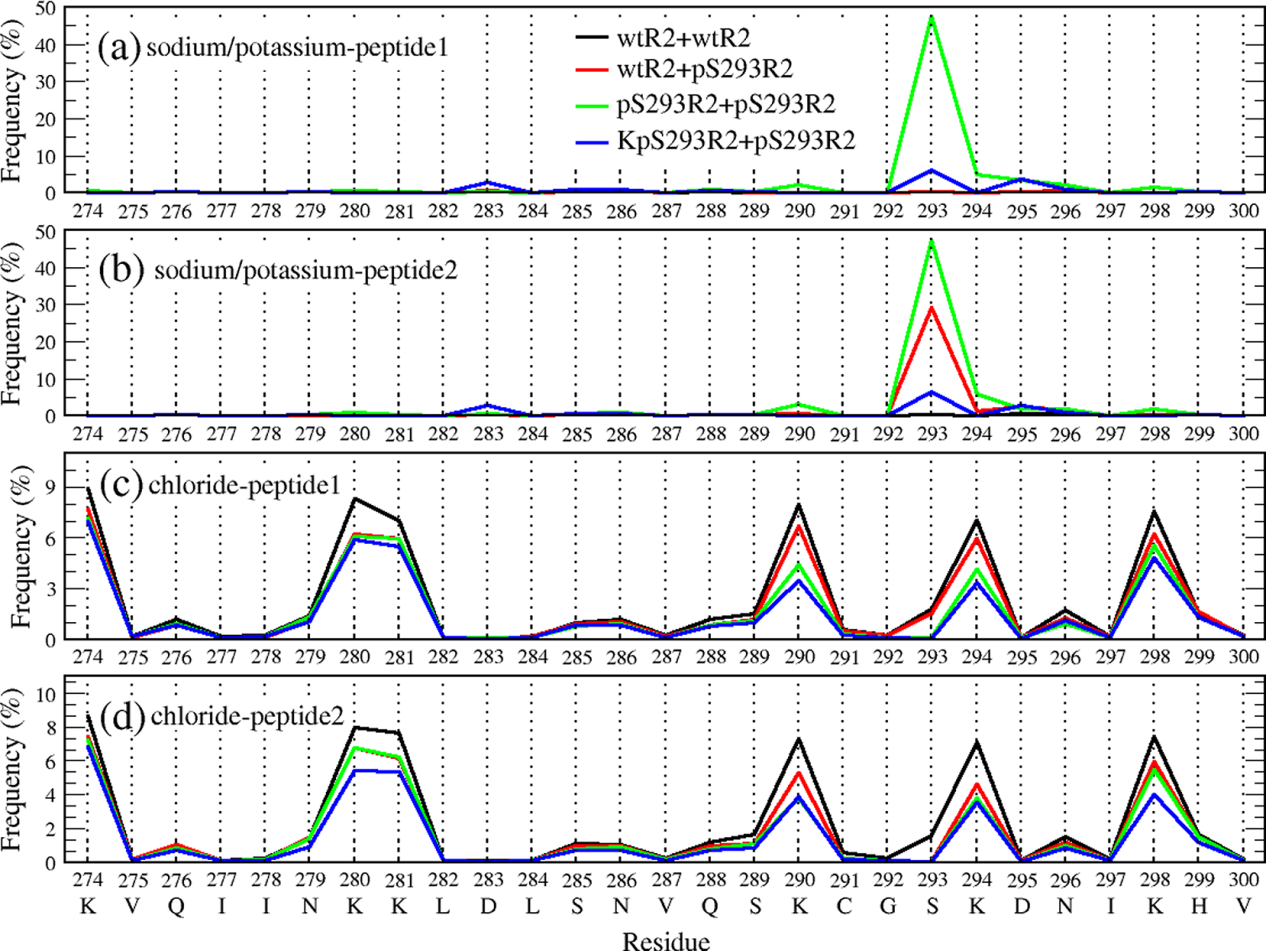
Ion–residue contact frequency (in %) in wtR2+wtR2 (black), wtR2+pS293R2 (red), pS293R2+pS293R2 (green), and KpS293R2+pS293R2 (blue) systems. The contact frequency of sodium (in the first three systems)/potassium (in KpS293R2+pS293R2 system) ions and the residues of the first and second peptides are shown in panels (a) and (b), respectively. The contact frequency of chloride ions and the residues of the first and second peptides are shown in panels (c) and (d), respectively.

**Figure 8:**
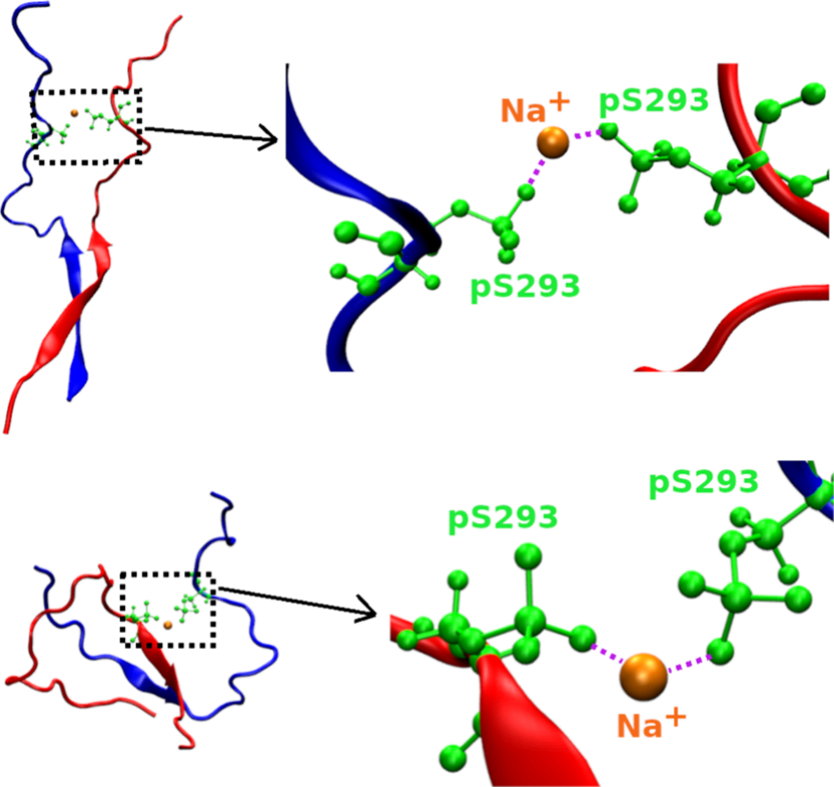
Two representative structures of pS293-Na+-pS293 bridges formed in the pS293R2+pS293R2 dimer.

Sodium and potassium are the most abundant cations in the brain with markedly different concentrations in cytosol (inside the neuron) and the extracellular fluid. In the cytosol, sodium concentration is around 12 mM, while potassium ranges from 130 to 150 mM. Conversely, in the extracellular fluid, sodium concentration is about 145 mM, and potassium is around 4 mM.^40^ Therefore, it is also of interest to study the interaction between potassium ions and the residues of pS293R2 peptides, particularly the pS293 residue, and to investigate how these interactions impact the oligomerization of pS293R2 peptides. Starting with the initial dimeric pS293R2+pS293R2 system, we replaced sodium ions with potassium ions to create a new dimeric system, KpS293R2+pS293R2. We then performed REMD simulations for this new dimeric system using the same protocol as that used for the original pS293R2+pS293R2 system. Our results indicated that although potassium ions interact more strongly with the pS293 residues than with other residues of the pS293R2 peptides, their interactions were still much weaker than those between sodium ions and the pS293 residues (Figure 7 and Figure S7). Furthermore, the pS293-K^+^-pS293 bridging interaction was not observed during the simulation. However, the overall structural parameters of the pS293R2 dimer in the KpS293R2+pS293R2 system, including β-content, SASA, Rg, and intermolecular residue–residue interactions, indicate that phosphorylation still enhances the oligomerization of R2 peptides (Table 2).

### Representative Structures of Monomeric and Dimeric Wild-Type and/or Phosphorylated-S293 R2 peptides

In a traditional docking screening, the target is usually taken from the crystal structures. For the case of amyloid aggregation, targets are fibril structures, not monomers nor soluble oligomers, which cannot be captured by experiments. However, amyloid aggregation is a dynamic process in which monomers aggregate into soluble oligomers, followed by fibril formation. Therefore, the docking screening of amyloid aggregation inhibitors will be more efficient if the targets include monomers and oligomers. On the other side, monomeric and oligomeric structures of amyloid peptides can be obtained from sampling MD simulations. In this MD simulation study, we also aimed to identify representative structures for the monomer and dimer of R2 peptides, which could be potential targets in the screening of tau oligomerization. We performed a clustering analysis on the sampled monomeric and dimeric R2 structures. Figure 9 shows the 10 representative structures, which are the centers of the 10 most populated clusters in each simulation system. The coordinates of the representative structures are included in SI.

**Figure 9:**
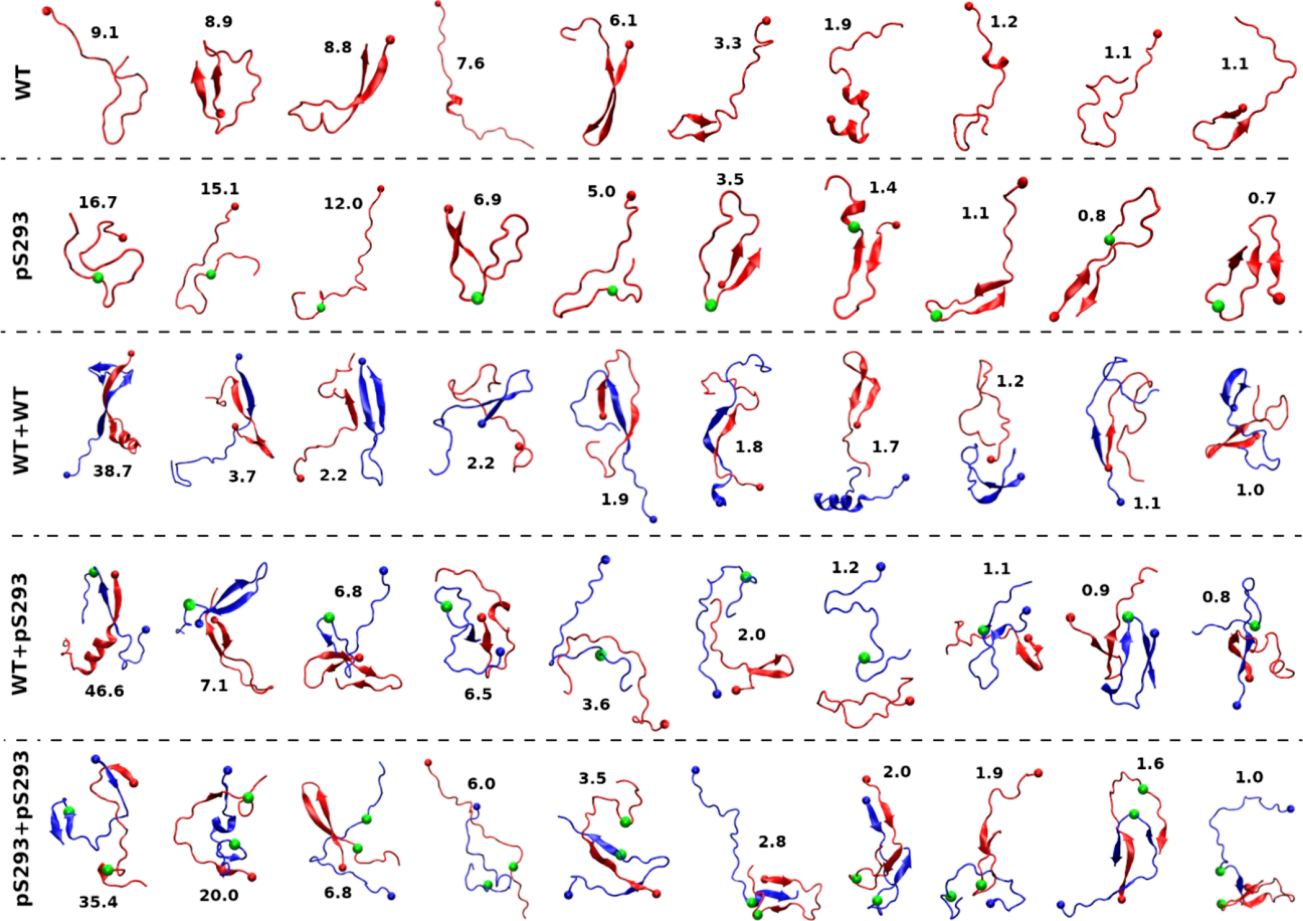
Representative structures of monomeric and dimeric R2 peptides.

## Discussion

In tauopathies such as AD, Tau with abnormal phosphorylation mislocates from MT-binding and aggregates into pathogenic forms, including soluble oligomers, insoluble fibrils, and NFTs.^3,6,41–43^ Although insoluble NFTs composed of insoluble fibrils are a hallmark of tauopathies, soluble oligomers, particularly low-weight ones such as dimers and trimers, are recognized as major contributors to tau pathologies.^4–6,42,43^ Thus, it is of great interest to investigate tau oligomerization regarding abnormal phosphorylation, to support the development of therapeutic agents targeting tau aggregation-related diseases. Even though TOs can be determined experimentally, it is still challenging to characterize the metastable structures of soluble TOs experimentally. Fortunately, MD simulations, which are capable of describing biomolecules at the atomic level, can complement experiments to address the challenge of investigating tau oligomerization. On the other hand, it takes considerable computational resources to perform all-atom MD simulation with explicit solvent for a large biomolecular system, such as oligomers of full-length tau. Therefore, some tau fragments such as tau repeats, which play a critical role in tau aggregation, are frequently used to represent the full-length tau to some degree in tau oligomerization studies.^29,34–36,44,45^

The tau R2 repeat, with residues ranging from 274 to 300, contains three serine residues, which can be phosphorylated. Among those three residues, phosphorylated S289 and S293 have been found in AD brain only, while phosphorylated S285 has not been discovered in healthy or AD brain. It is pointed out that phosphorylated S289 (pS289) and S293 (pS293) are considered to have abnormal phosphorylation. Recently, we investigated the impact of phosphorylated S289 on the oligomerization of tau R2 repeat peptide.^29^ Our result demonstrated that phosphorylation at S289 significantly impacted on the monomeric and dimeric structures of R2 repeat and promoted the oligomerization of R2 repeat. In this work, we expanded our study to the phosphorylation at S293. We found that pS293 also promoted tau R2 repeat oligomerization. The phosphorylation at S293 increased the intramolecular interaction and the compactness of monomers, the intermolecular interactions, the compactness, and the β-sheet formation of dimers. The average values of *R*_g_ of pS289R2 and pS293R2 monomer, 1.25 and 1.26 nm correspondingly, are larger than that of wtR2. The triad bridge of pSer-Na^+^-pSer, which was observed for both pS289 and pS293 dimer, plays an important role in the oligomerization of the R2 peptide. However, our detailed analysis suggested that the impact of phosphorylation on monomeric and dimeric R2 peptides is significantly different for the two serine residues. For the monomeric peptide, pS293 increased β-sheet content and reduced coil and helix contents, while pS289 promoted turn content and decreased the helix content. Moreover, pS293 increased the β-propensity of residues at 280, 281, and 284–286 and particularly C-terminal ones (296–299). In contrast, p289 increased β-propensity of residues at 282–284, 293, and 294, but decreased the β-propensity of N-terminal residues (275–279) (Figure S8). Our data indicated that pS289 supported ordered–disordered transition, while pS293 supported the disordered–ordered structural transition of R2 repeat monomers (Figure S8). For the dimeric peptide, although the average secondary structural contents were similar for pS293 and pS289 dimers, their residue-based secondary structural profiles were different (Figure S9). Interestingly, in comparison with the wild-type peptide, pS289 peptides presented higher β propensities at N-terminal residues, while pS293 demonstrated higher β propensities at residues 289–291. In both monomeric and dimeric cases, the pS293 systems have smaller *R*_g_ and SASA than those of the pS289 systems, indicating that the interaction increase due to phosphorylation is a little bit stronger at S293 than at S289.

Sodium and potassium concentrations differ substantially between the cytosol and extracellular fluid, and the Na^+^/K^+^ ratio is significantly elevated in the brains of individuals with AD.^46,47^ Clinical studies have linked high dietary salt with tau phosphorylation and cognitive impairment,^48^ whereas higher potassium intake has been associated with neuroprotective effects.^49^ Previous experiments have shown that cations, particularly divalent ions such as Zn^2+^, Mg^2+^, and Ca^2+^, promote tau hyperphosphorylation and induce tau aggregation.^39,50^ However, the effects of sodium and potassium on the aggregation of phosphorylated tau have not yet been explored. In this work, in addition to investigating the impact of phosphorylation at S293 on the aggregation of tau R2 peptides, we also examined how sodium and potassium ions influence the oligomerization of pS293R2 peptides. Our simulations reveal that Na^+^, but not K^+^, stabilizes a pS-Na^+^-pS bridge, likely due to its stronger hydration-free energy, smaller ionic radius, and slower diffusion, which collectively favor tighter binding to phosphate groups.^51–53^ In contrast, K^+^ interacts more weakly and fails to form a stable pS-K^+^-pS bridge. Compared to these monovalent ions, divalent cations such as Ca^2+^, Zn^2+^, and Mg^2+^ possess even higher charge densities and stronger hydration,^51–53^ suggesting they may more readily form pS-cation-pS bridges. These findings suggest that phosphorylation-driven oligomerization is robust across various ionic environments, with Na^+^– and potentially divalent cation-mediated bridging interactions providing an additional stabilizing mechanism.

## Conclusions

Aberrant phosphorylation is a hallmark of tauopathies, but its atomistic role in promoting oligomerization can be elucidated only through MD simulations. In this study, we performed extensive REMD simulations to investigate the effect of S293 phosphorylation on the monomeric and dimeric structures of tau R2 repeat peptides. Our results demonstrate that S293 phosphorylation enhances oligomerization by strengthening intermolecular interactions, consistent with our previous findings for S289. Both phosphorylation sites promote β-sheet formation and structural compactness, but they exert distinct effects on the secondary structure: pS289 facilitates an ordered-to-disordered transition, whereas pS293 induces a disordered-to-ordered transition. Overall, pS293 shows a capacity greater than that of pS289 to promote R2 peptide oligomerization. We further examined the interactions between phosphorylated residues and the cytosolic cations. A pS-cation-pS triad bridge was observed with sodium but not with potassium, reflecting their distinct physicochemical properties. In light of this, and considering the known effects of divalent cations such as Zn^2+^ and Ca^2+^ on phosphorylated tau aggregation, our findings suggest that these triad bridges may play a role in promoting phosphorylated tau oligomerization. It should be noted that our simulations were conducted on a 27-residue fragment of the R2 repeat rather than on full-length tau. While fragment-based models provide valuable atomistic insights into local, sequence-specific effects of phosphorylation, they cannot capture the full complexity of tau aggregation *in vivo*, where cofactors such as RNA, multiple post-translational modifications, and inter-repeat interactions play crucial roles. Therefore, although our results indicate that pS293 promotes oligomerization of R2 peptides more strongly than does pS289, future studies involving full-length tau and relevant cofactors will be necessary to fully establish the role of S293 phosphorylation in tau aggregation and pathology.

## Materials and Methods

### Simulation Details

To prepare initial structures for the replica exchange MD simulations of monomeric and dimeric systems, we constructed monomeric structural data banks in the following steps. Tau R2 peptide was taken from an experimental structure of R2-microtubule complex (PDB code 6CVN).^54^ The peptide named wtR2 was capped by acetyl (ACE) at the *N*-terminus and *N*-methylamine (NME) at the C-terminus. wtR2 was placed into an octahedron box of explicit water, and the smallest distance of the box border to the peptide is larger than 1.2 nm. This system, wtR2 in water, underwent 500 ns NPT simulation at a pressure of 1 atm and temperature of 300 K. A Gromos clustering analysis with a cutoff of 0.35 nm was performed for the peptide structures collected from the last 400 ns of the 500 ns NPT simulation. The conformations in the 80 largest clusters were deposited in the monomeric structural data bank. For the phosphorylated S293 R2 peptide (pS293R2), it was obtained by phosphorylating the experimental R2 peptide at the S293 residue. Similar steps were performed to prepare a monomeric structural data bank for the pS293R2 peptide. The structural data banks were used to generate initial structures of the replica exchange MD simulation.

In total, we built two REMD monomeric systems, the wild-type R2 (wtR2) and the phosphorylated-S293 R2 (pS293R2), and three REMD dimeric systems, namely, the wild-type R2 peptide, wtR2+pS293R2 with S293 in one peptide being phosphorylated, and pS293R2+pS293R2 with S293 in both peptides being phosphorylated. For each REMD monomeric system, 42 simulation systems were prepared. Specifically, a monomer was randomly selected from the monomeric structural data bank and then centered in an octahedron sized to 7.5 nm. Then, 10,300 explicit water molecules were added to the simulation box. For each REMD dimeric system, 49 simulation systems were prepared. Specifically, two randomly selected monomers in the monomeric structural data bank were placed in an octahedral box size of 8.1 nm. The two monomers had a distance between 0.13 and 0.33 nm; 13,000 explicit water molecules were added to the simulation box. Using box sizes of 7.5 nm for monomeric systems and 8.1 nm for dimeric systems guarantees that the shortest distance between any peptide atom and the water box boundary is no less than 1 nm. For a monomeric or dimeric simulation system, sodium/potassium cations (Na^+^/K^+^) and chloride anions (Cl^–^) were added to the simulation system until the system was neutralized and the salt concentration was about 0.15 M.

GROMACS 2018 package^55^ was employed for all simulations. The peptides were described by Charmm36m force field,^56^ which is currently the most suitable to simulate amyloid aggregations based on a series of benchmarking simulation tests.^57,37^ The TIP3P model^58^ was used to represent explicit water molecules. The parameters of the ions were taken from Beglow and Roux’s work for K+ and Cl–,^59^ and from Noskov and Roux’s work for Na+,^60^ which well reproduced experimental results.^61^ The solvated systems were minimized using the steepest descent method and were equilibrated for 1 ns at a constant pressure of 1 atm. The pressure and temperature of the simulations were controlled using the Berendsen coupling method^62^ with a relaxation time of 3 ps and the Bussi-Donadio-Parrinello velocity scaling method^63^ with a relaxation time of 1 ps, respectively. The equations of motion were integrated using a leapfrog algorithm^64^ with a time step of 2 fs (fs). 300 and 500 ns NPT sampling simulations were subsequently conducted for each replica of each monomeric and dimeric system, respectively. The above simulation protocols resulted in 12.6 and 24.5 μs (μs) of simulations for each monomeric and dimeric system and 98.7 μs in total for all five systems. The LINCS algorithm^65^ was used to constrain the lengths of all covalent bonds. The van der Waals forces were calculated with a cutoff of 10 Å, and the particle mesh Ewald method^66^ was employed to treat the long-range electrostatic interactions. The nonbonded interaction pair list was updated every 5 fs using a cutoff of 10 Å. Periodic boundary conditions were applied to all of the simulations. The REMD temperatures ranged from 300 to 400 K (Table S1 in Supporting Information). Exchanges between two replicas were attempted every 2 ps, leading to a mean acceptance ratio of 18%.

### Data Analysis

The structures of R2 monomers and dimers sampled by MD simulations were characterized by intramolecular and intermolecular side chain–side chain contacts, intramolecular and intermolecular backbone hydrogen bonds (H-bonds), solvent-accessible surface areas (SASAs), gyration of radius (*R*_g_), and secondary structural contents. A side chain–side chain contact was formed if the minimum distance between two residue side chains was within 4.5 Å. The distance between two residues was the minimum distance between any atom of the first residue to any atom of the second residue. An H-bond is formed if the acceptor–donor distance was within 3.5 Å and the acceptor–donor-H angle was less than 30°. The intramolecular interactions were calculated for two residues *i* and *j* when they are not nearest neighbors. The secondary structural contents were calculated by using the STRIDE algorithm (with helix content including 3–10 helix, π-helix and α-helix),^67,68^ and H-bond, SASA, and *R*_g_ were calculated using GROMACS tools. The linkage clustering method was applied with a cutoff of 0.45 nm for monomers and 0.8 nm for dimers.

## Supplementary Material



## Data Availability

All input files, force field parameters, and representative trajectories generated in this study have been deposited on Zenodo and are accessible at 10.5281/zenodo.17093145.

## Notes

REMD simulation information (Table S1); time dependence of RMSD of monomeric and dimeric R2 peptides (Figure S1); time dependence of *R*_g_, SASA, and β-content of monomeric and dimeric R2 peptides (Figures S2 and S3); distributions of *R*_g_, SASA, number of intramolecular (*N*_intra‑SC_) interaction of monomers, and number of intermolecular side chain–side chain interaction (*N*_inter‑SC_) of dimeric R2 peptides (Figure S4); secondary structures, SASA, and *R*_g_ of R2 monomers at different temperatures (Figure S5); secondary structural contents, SASA, and *R*_g_ of R2 dimers at different temperatures (Figure S6); populations of ion-Ser/pSer distance (Figure S7); secondary structural differences along residues of pS289R2 and pS293R2 peptides to the wild-type one (Figure S8); secondary structural differences along residues of wt + pS289, pS289 + pS289, wt + pS293, and pS293 + pS293 dimer peptides to wild-type dimers (Figure S9) (PDF)
