# Supplementary material for: Aberrant S293 Phosphorylation Drives Oligomerization of Tau Repeat R2: Insights from Molecular Dynamics Simulations
Source: ACS Chem Neurosci. 2025 Oct 23;16(21):4297–308. doi: 10.1021/acschemneuro.5c00734 (PMC12593403; doi:10.1021/acschemneuro.5c00734)
Supplement: Supplementary file 1 [file cn5c00734_si_001.pdf]

Aberrant S293 Phosphorylation Drives Oligomerization of Tau Repeat R2: Insights from Molecular Dynamics Simulations

[SUPPORTING INFORMATION]

Viet Hoang Man,[1*] Xibing He,[1] Phuong H. Nguyen[2], Jie Gao,[3] and Junmei Wang[1*]

[1] *Department of Pharmaceutical Sciences and Computational Chemical Genomics Screening Center, School of Pharmacy, University of Pittsburgh, Pittsburgh, PA 15261, USA.*

[2]*Laboratoire de Biochimie Theorique UPR 9080, CNRS, Universit´e Denis Diderot, Sorbonne Paris Cit´e IBPC, 13´Rue Pierre et Marie Curie, 75005 Paris*

[3] *Department of Neuroscience, The Ohio State University Wexner Medical Center, Columbus, OH 43210, USA.*

[*]E-Mails:

Viet Hoang Man: vhm3@pitt.edu

Junmei Wang: junmei.wang@pitt.edu

Table S1. Parameters of replica exchange molecular dynamics (REMD) simulations. The parameters include replica temperatures of monomeric systems (MoSys) and dimeric systems (DiSys), and the exchange rates. The standard deviation of all exchange rates is around 0.01

Replica temperatures			Exchange rate					
Rindex	MoSys	DiSys	Exchange pair	Systems				
				wtR2	pS293R2	wtR2-+wtR2	wtR2+ pS293R2	pS293R2+ pS293R2
0	298	298						
1	300.12	299.87	0 – 1	0.19	0.19	0.19	0.19	0.19
2	302.26	301.75	1 – 2	0.18	0.18	0.19	0.19	0.19
3	304.42	303.65	2 – 3	0.18	0.18	0.18	0.18	0.18
4	306.6	305.56	3 – 4	0.18	0.18	0.19	0.19	0.19
5	308.79	307.48	4 – 5	0.18	0.19	0.19	0.19	0.19
6	311	309.42	5 – 6	0.18	0.18	0.19	0.19	0.18
7	313.23	311.37	6 – 7	0.18	0.18	0.18	0.18	0.18
8	315.48	313.34	7 – 8	0.18	0.18	0.18	0.18	0.18
9	317.75	315.32	8 – 9	0.18	0.18	0.18	0.18	0.18
10	320.03	317.32	9 – 10	0.18	0.18	0.18	0.18	0.18
11	322.33	319.32	10 – 11	0.18	0.18	0.18	0.18	0.18
12	324.65	321.35	11 – 12	0.18	0.18	0.18	0.18	0.18
13	326.99	323.38	12 – 13	0.18	0.17	0.18	0.18	0.18
14	329.34	325.43	13 – 14	0.18	0.18	0.18	0.18	0.18
15	331.72	327.5	14 – 15	0.18	0.17	0.18	0.18	0.18
16	334.11	329.57	15 – 16	0.18	0.17	0.18	0.18	0.18
17	336.51	331.66	16 – 17	0.18	0.18	0.18	0.18	0.18
18	338.94	333.77	17 – 18	0.17	0.17	0.18	0.18	0.18
19	341.38	335.89	18 – 19	0.18	0.17	0.18	0.18	0.18
20	343.84	338.02	19 – 20	0.17	0.17	0.18	0.18	0.18
21	346.31	340.16	20 – 21	0.18	0.17	0.18	0.18	0.18
22	348.8	342.32	21 – 22	0.17	0.17	0.18	0.18	0.18
23	351.31	344.49	22 – 23	0.17	0.17	0.18	0.18	0.18
24	353.84	346.67	23 – 24	0.17	0.18	0.18	0.18	0.18
25	356.38	348.87	24 – 25	0.17	0.17	0.18	0.18	0.17
26	358.94	351.08	25 – 26	0.17	0.17	0.18	0.18	0.18
27	361.51	353.3	26 – 27	0.17	0.17	0.18	0.18	0.18
28	364.1	355.54	27 – 28	0.17	0.17	0.17	0.17	0.17
29	366.7	357.78	28 – 29	0.18	0.17	0.18	0.18	0.18
30	369.32	360.04	29 – 30	0.17	0.17	0.18	0.18	0.17
31	371.96	362.32	30 – 31	0.17	0.17	0.17	0.17	0.17
32	374.61	364.6	31 – 32	0.18	0.17	0.18	0.18	0.18
33	377.28	366.9	32 – 33	0.17	0.17	0.17	0.17	0.17
34	379.96	369.21	33 – 34	0.17	0.17	0.18	0.18	0.18
35	382.65	371.53	34 – 35	0.17	0.17	0.18	0.18	0.18
36	385.36	373.86	35 – 36	0.17	0.17	0.18	0.18	0.18
37	388.09	376.2	36 – 37	0.17	0.17	0.17	0.17	0.18
38	390.82	378.56	37 – 38	0.17	0.18	0.17	0.17	0.17
39	393.58	380.93	38 – 39	0.17	0.17	0.17	0.17	0.18
40	396.34	383.31	39 – 40	0.18	0.17	0.17	0.17	0.17
41	399.12	385.7	40 – 41	0.19	0.19	0.18	0.18	0.18
42		388.1	41 – 42	0.19	0.19	0.18	0.18	0.17
43		390.51	42 – 43			0.18	0.18	0.18
44		392.93	43 – 44			0.18	0.18	0.18
45		395.36	44 – 45			0.18	0.18	0.18
46		397.81	45 – 46			0.17	0.17	0.18
47		400.26	46 – 47			0.18	0.18	0.18
48		402.73	47 – 48			0.19	0.19	0.19

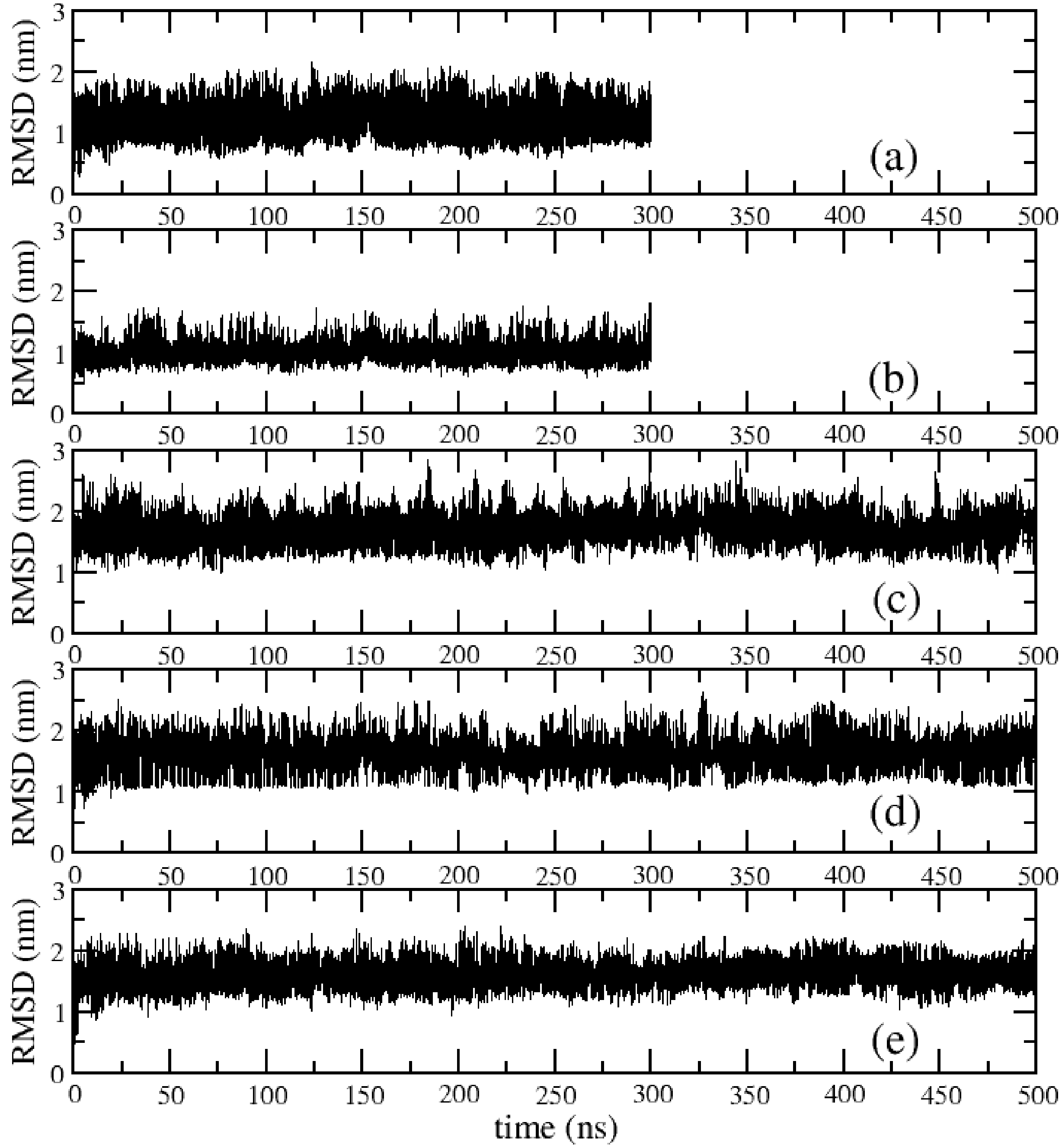

Figure S1: Time dependence of root mean square deviation (RMSD) of wtR2 (a), pS293R2 (b), wtR2+wtR2 (c), wtR2+pS293R2 (d), and pS293R2+pS293R2 (e) systems.

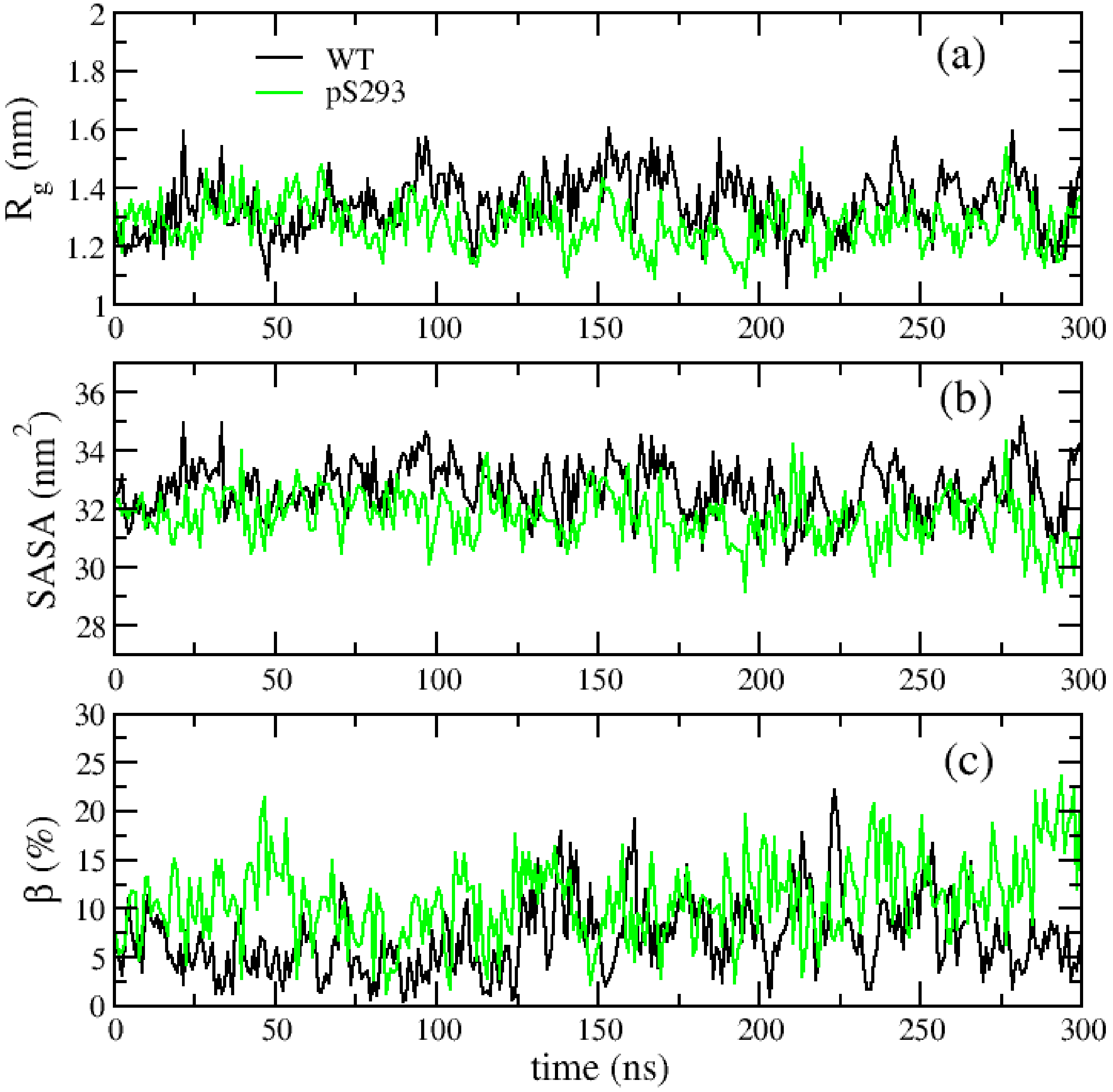

Figure S2: Time dependence of radius of gyration (Rg), solvation accessible surface area (SA) and β content of the monomeric peptides in wtR2 (black lines) and pS293R2 (green lines) systems. The data is averaged for every ns.

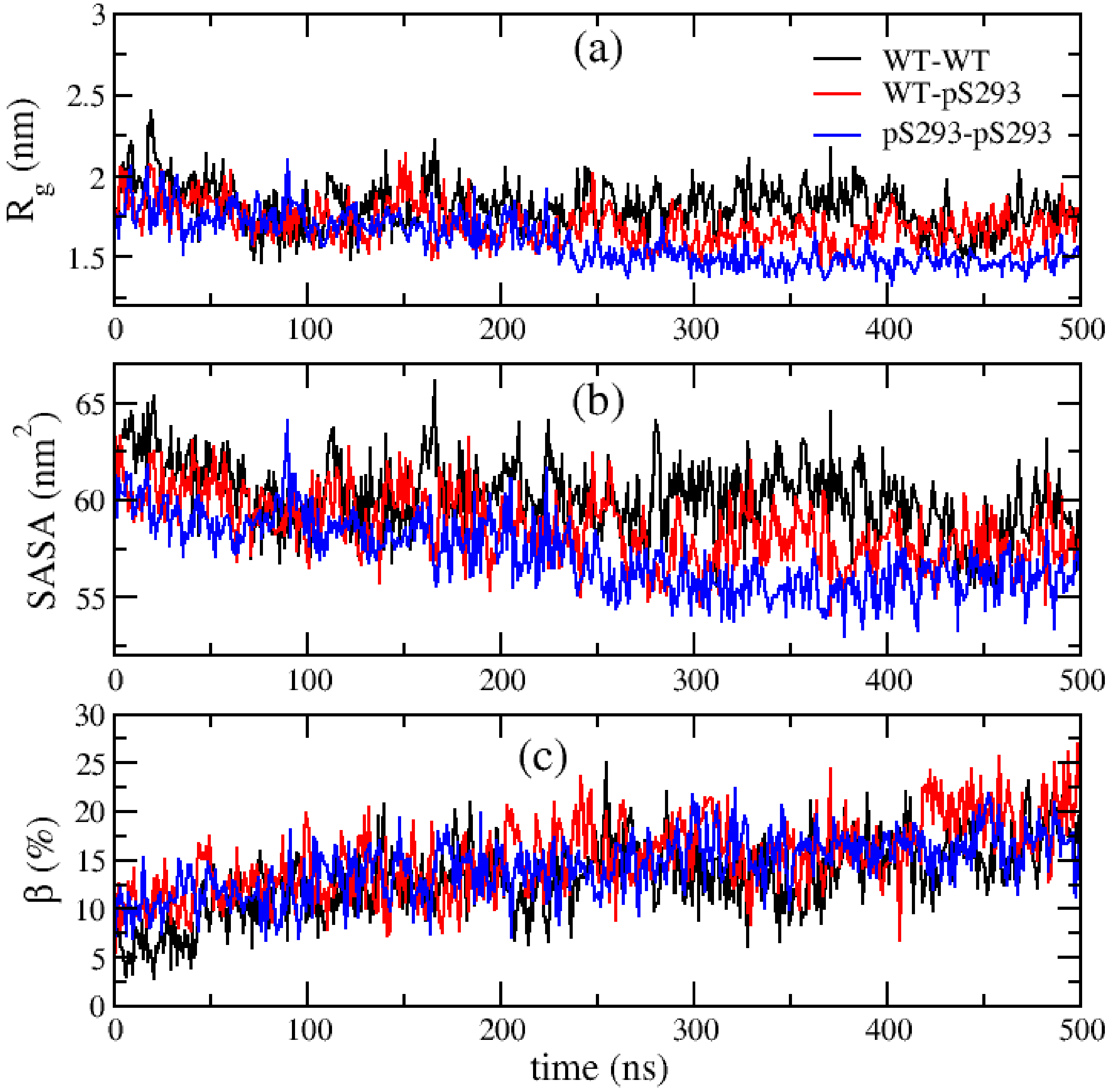

Figure S3: Time dependence of radius of gyration (Rg), solvent accessible surface area (SA) and β content of the dimeric peptides in wtR2+wtR2 (black lines) and wtR2+pS293R2 (red lines) and pS293R2+pS293R2 (blue lines) systems. The data is averaged for every ns.

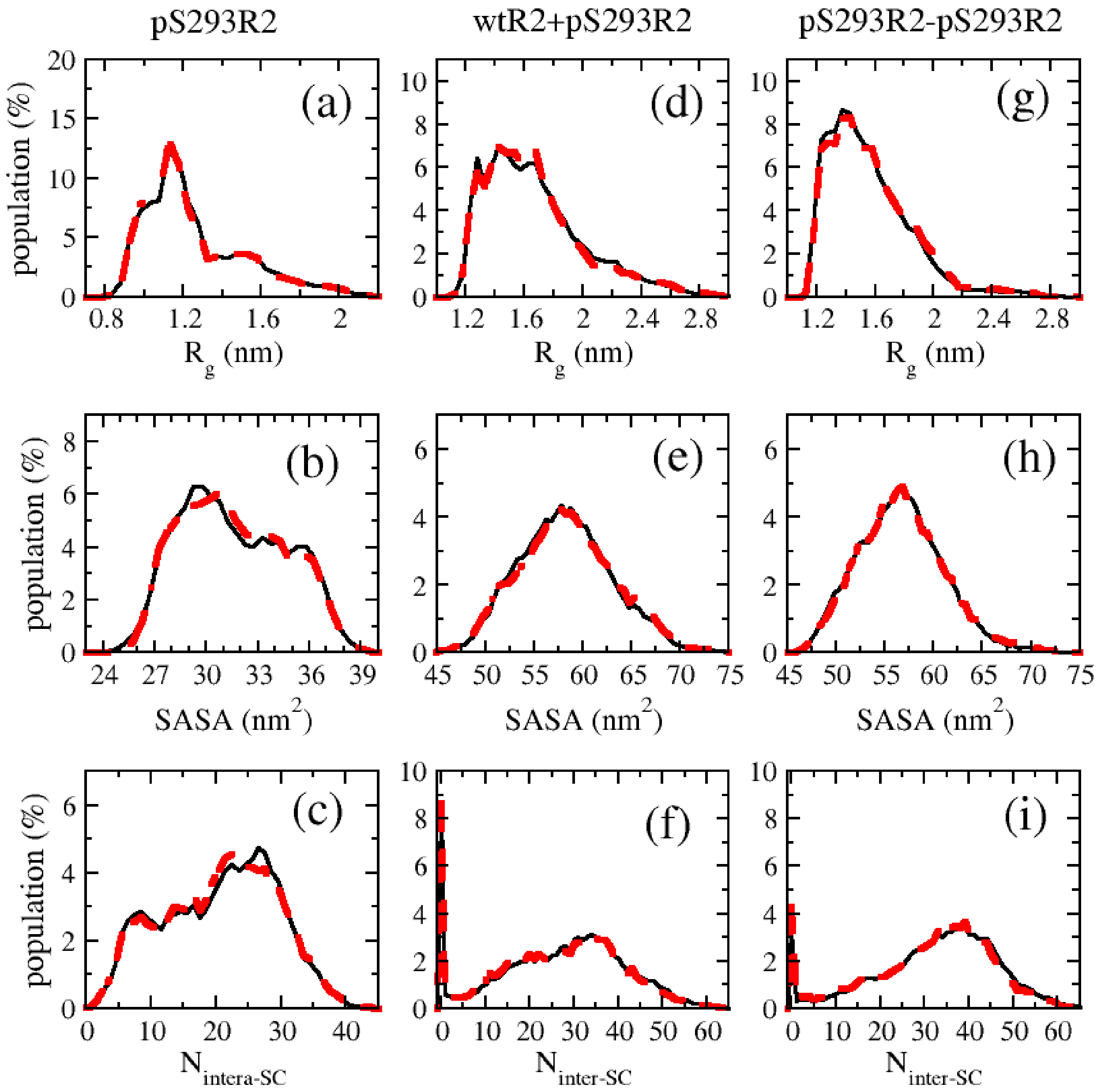

Figure S4: The distributions of gyration (Rg), solvent accessible surface area (SASA), number of intramolecular side chain-side chain interaction ($N_{intra\text{-}SC}$), and number of intermolecular side chain-side chain interaction ($N_{inter\text{-}SC}$) of pS293R2 monomer, WT+pS293 dimer, and pS293+pS293. For the monomer (a-c), the results were obtained from two ensemble statistics at 311 K replica: with 150 ns spanning from 100 ns to 250 ns (red dashed lines); with the last 200 ns (from 100 ns to 300 ns) (black solid lines). For the dimers, the results were obtained from two ensemble statistics at 309.4 K replica: with 300 ns spanning from 100 ns to 400 ns (red dashed lines); with the last 400 ns (from 100 ns to 500 ns) (black solid lines).

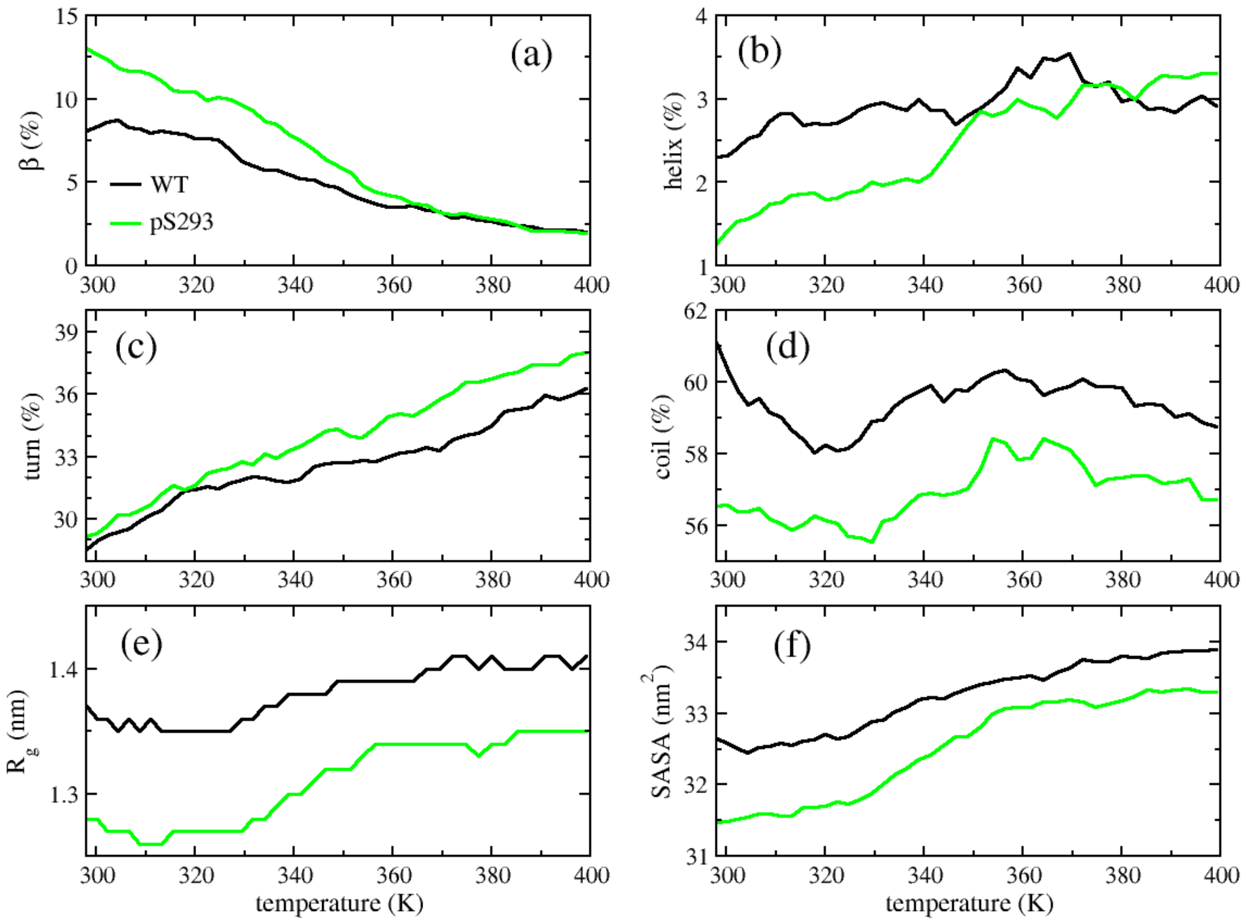

Figure S5: The β content (a), helix content (b), turn content (c), coil content (d), radius of gyrate (R_g) (e), and solvent accessible surface area (SASA) (f) of wild type (black lines) and pS293 (green lines) R2 monomers at different temperatures. The data is from the last 200 ns of each related replica.

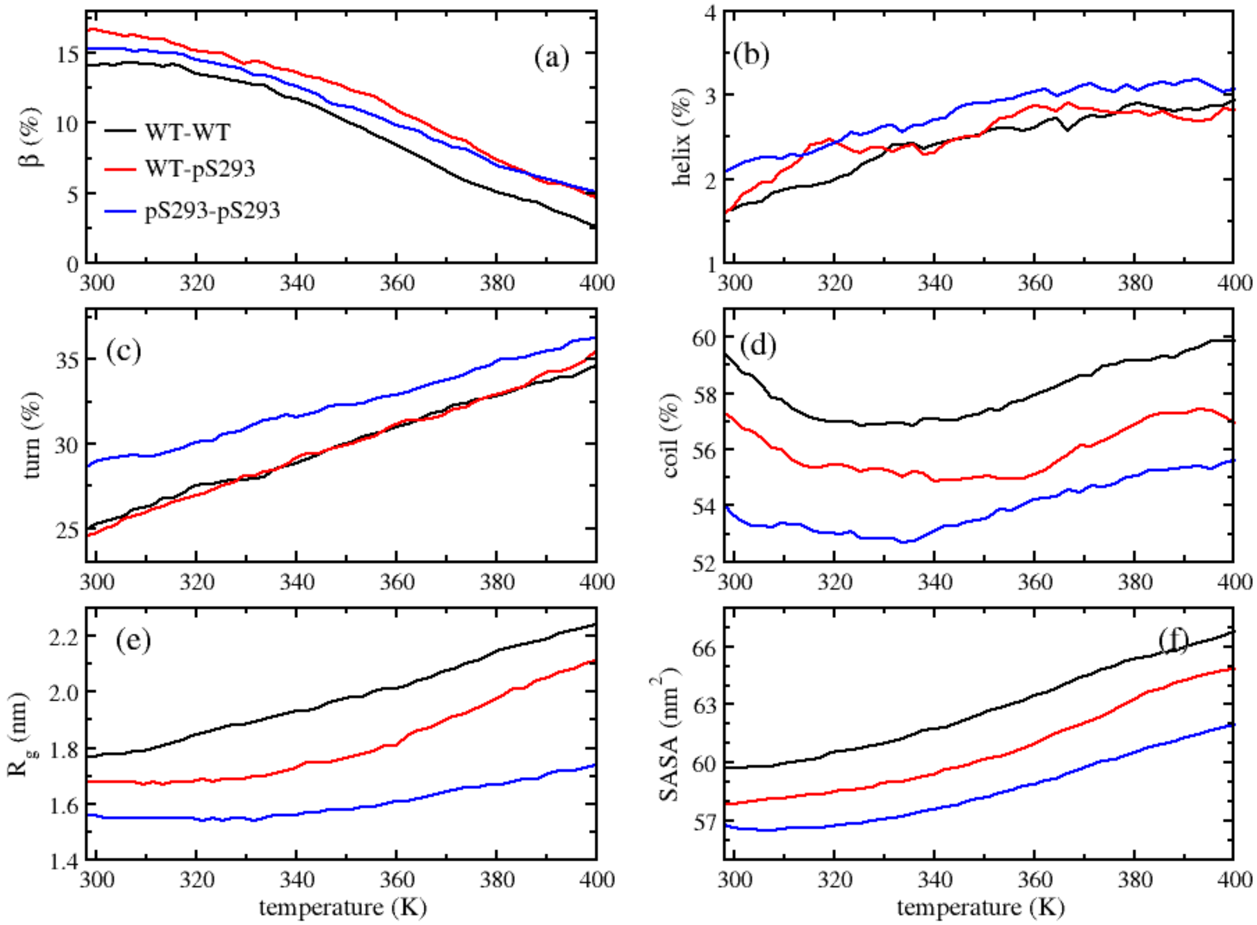

Figure S6: The β content (a), helix content (b), turn content (c), coil content (d), radius of gyrate (R_g) (e), and solvent accessible surface area (SASA) (f) of R2 dimers at different temperatures of REMD simulations. The data is from the last 400 ns of each related replica.

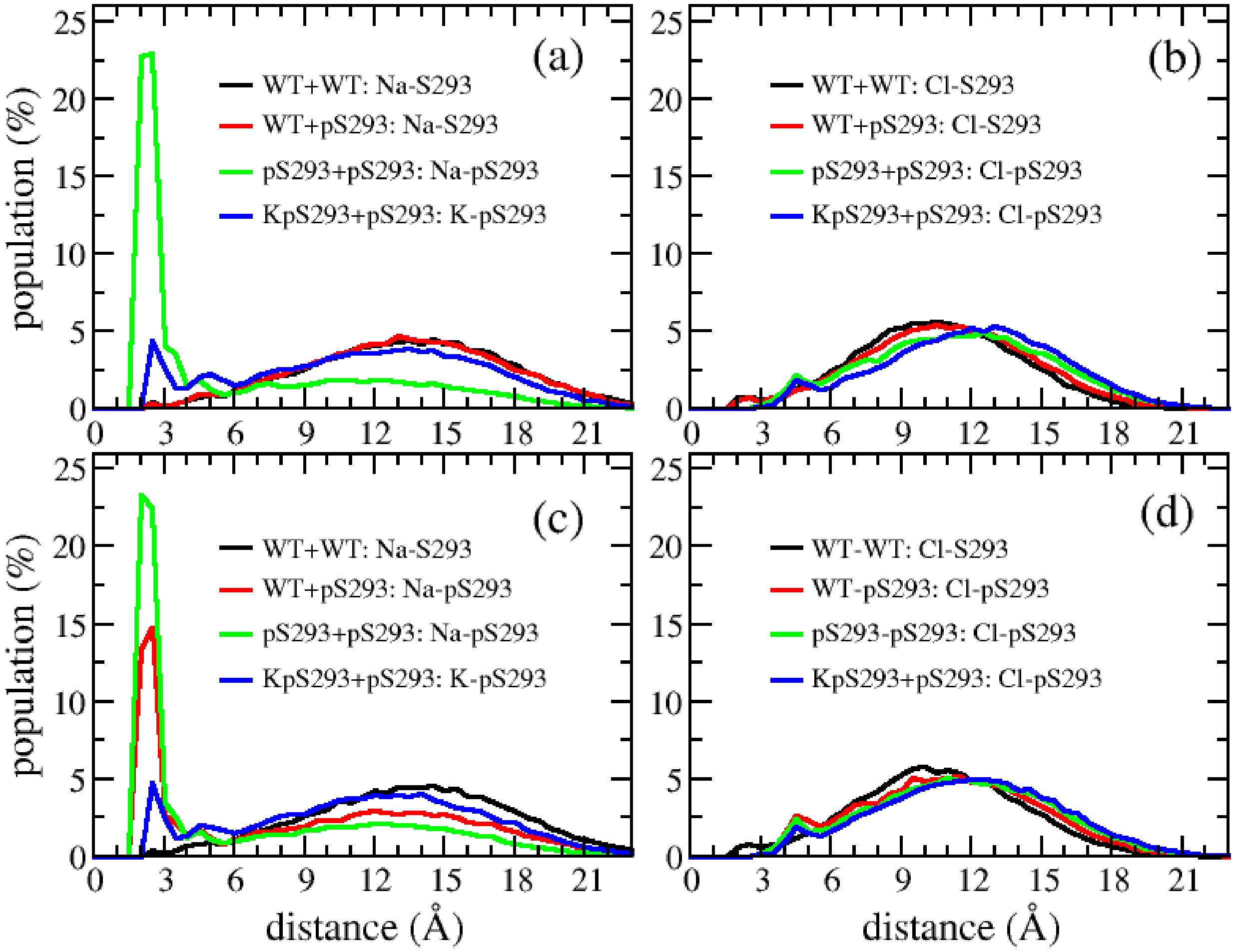

Figure S7: The populations of ion-Ser/pSer distance in WT-WT (black lines), WT-pS293 (red lines), and pS293-pS293 (green lines) dimeric systems. (a) distances between sodium ions and the first R2 peptide, (b) distances between chloride ions and the first R2 peptide, (c) the distances between sodium ions and the second R2 peptide, (d) the distances between chloride ions and the second R2 peptide.

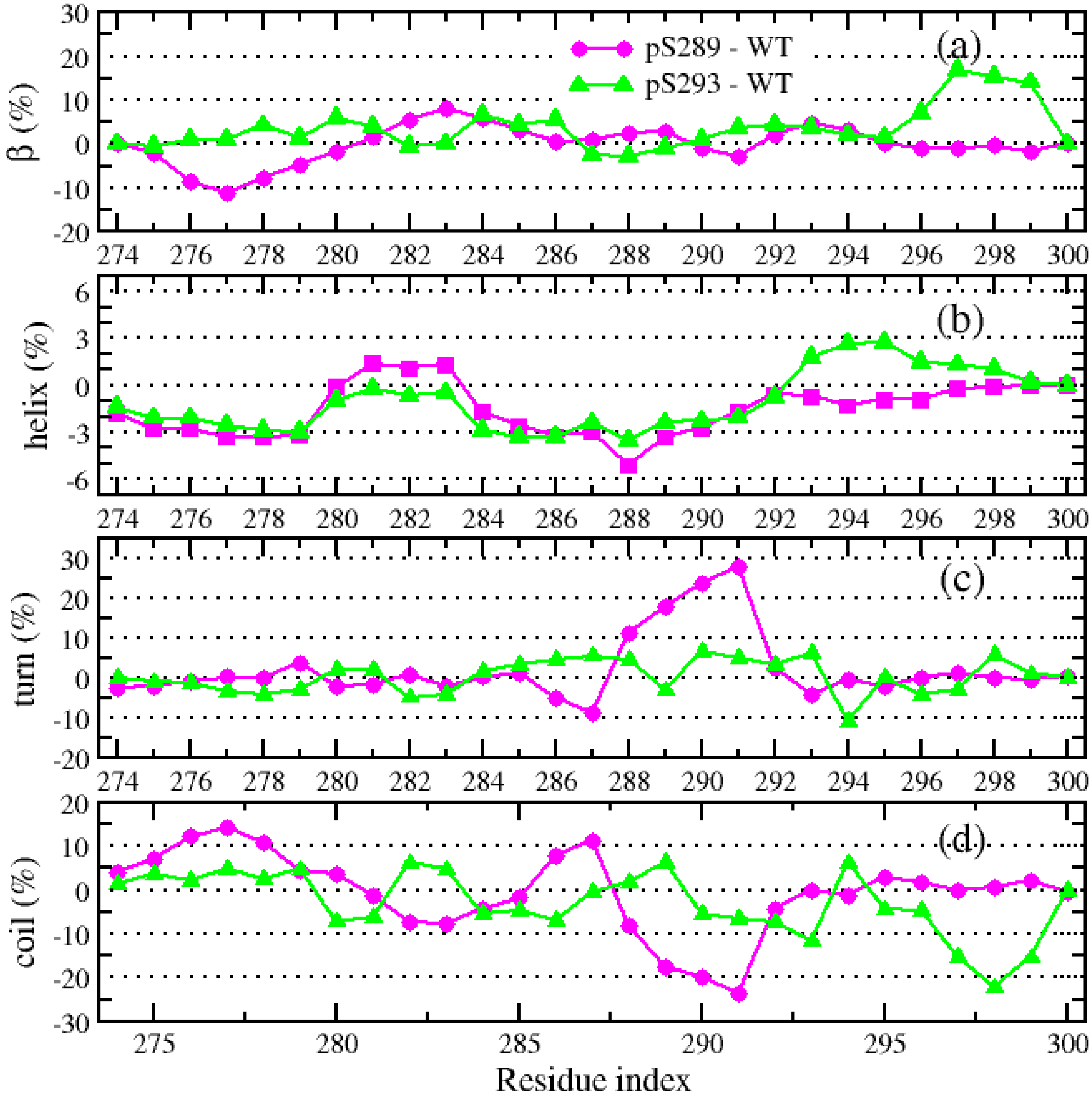

Figure S8: The secondary structural differences along residues of pS289R2 and pS293R2 peptides to wild-type one.

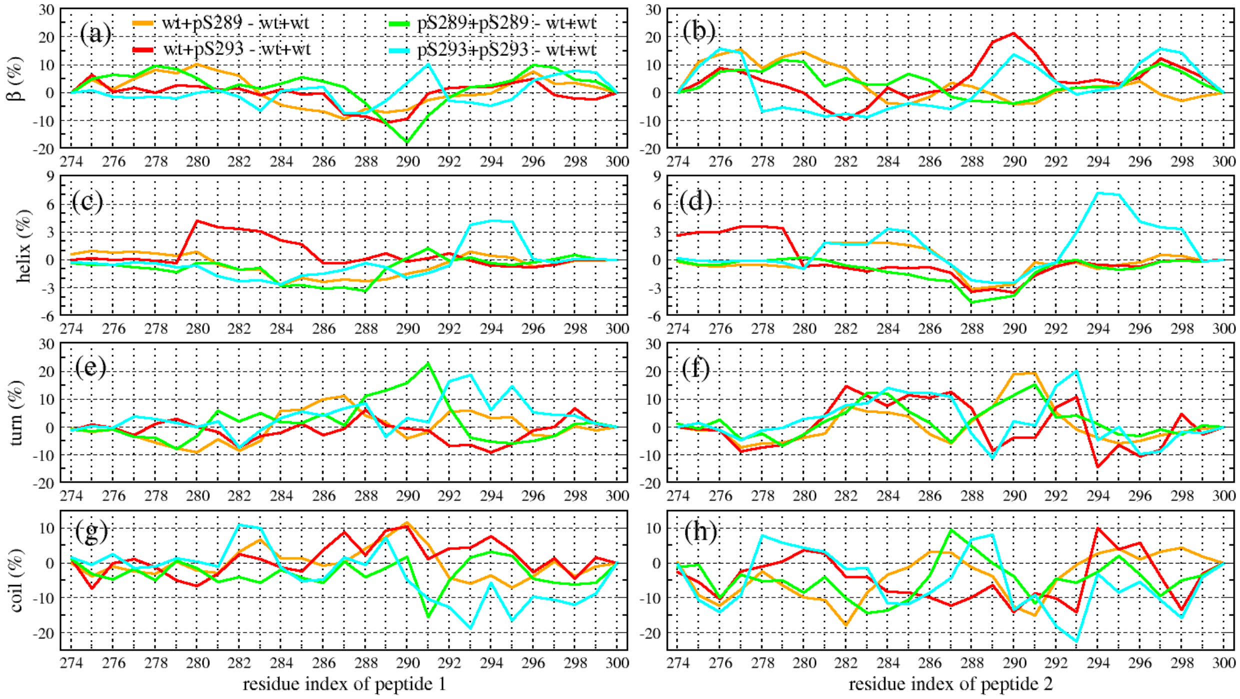

Figure S9: The secondary structural differences along residues of wt+pS289, pS289+pS289, wt+pS293 and pS293+pS293 dimers peptides to wild-type dimer.
